# Human Hematopoietic Stem/Progenitor Cells in Type One Diabetes Mellitus Treatment: Is There an Ideal Candidate?

**DOI:** 10.3390/cells12071054

**Published:** 2023-03-30

**Authors:** Ermes Carulli, Giulio Pompilio, Maria Cristina Vinci

**Affiliations:** 1 Doctoral Programme in Translational Medicine, Università di Milano, 20122 Milan, Italy; 2 Dipartimento di Scienze Cliniche e di Comunità, Università di Milano, 20122 Milan, Italy; 3 National Heart and Lung Institute, Imperial College London, London SW7 2BX, UK; 4 Unit of Vascular Biology and Regenerative Medicine, Centro Cardiologico Monzino IRCCS, 20138 Milan, Italy; giulio.pompilio@cardiologicomonzino.it (G.P.); cristina.vinci@cardiologicomonzino.it (M.C.V.); 5 Dipartimento di Scienze Biomediche, Chirurgiche e Odontoiatriche, Università di Milano, 20122 Milan, Italy

**Keywords:** T1DM, diabetes, CD34, HSPCs, AHST, cell therapy, cardiovascular disease

## Abstract

Type 1 diabetes mellitus (T1DM) is a highly prevalent autoimmune disease causing the destruction of pancreatic islet β-cells. The resulting insulin production deficiency leads to a lifelong need for insulin re-placement therapy, systemic complications, and reduced life quality and expectancy. Cell therapy has been extensively attempted to restore insulin independence (IID), and autologous nonmyeloablative hematopoietic stem cell transplantation (AHST) has appeared to give the most promising results, but with a highly variable quote of patients achieving IID across the studies. We performed a comprehensive review of the trials involving stem cells, and in particular AHST, for the treatment of T1DM. We then pooled the patients enrolled in the different trials and looked for the patient characteristics that could be associated with the achievement of IID. We found a significantly higher probability of achieving IID in older patients (OR 1.17, 95%CI 1.06–1.33, *p* = 0.002) and a significantly lower probability in patients with a history of ketoacidosis (OR 0.23, 95%CI 0.06–0.78, *p* = 0.023). This suggests that there could be a population of patients more likely to benefit from AHST, but further data would be required to depict the profile of the ideal candidate.

## 1. Introduction

Type 1 diabetes mellitus (T1DM) is an autoimmune disease affecting pancreatic islets and, in particular, β-cells, which are responsible for insulin production [1,2]. Data from a metanalysis performed in 2020 showed a worldwide incidence of 15 per 100,000 people and a prevalence of 9.5%, with a constantly increasing trend [3]. Moreover, T1DM has been found to account for 5.6% of diabetes cases in an American National Health Interview Survey [4]. Its incidence rate peaks at 10–14 years, and only one quarter of cases are diagnosed in adults [5]. Complications are mostly driven by hyperglycaemia and include microvascular (retinopathy, nephropathy, neuropathy) and macrovascular (cardiovascular disease, cerebrovascular accidents, peripheral vascular disease) damage. However, unlike type 2 diabetes mellitus (T2DM), T1DM complications can extend beyond the common effects of hyperglycaemia: for example, cardiac autoimmunity and cardiac autoantibodies have been found to be associated with poor glycaemic control in T1DM but not in T2DM, with a pathogenetic model more similar to Chagas cardiomyopathy [6]. On the other hand, hyperglycaemia in T2DM is due to a combined mechanism of insulin resistance and insulin production exhaustion; T2DM tends to onset at an older age and is usually associated with other cardiovascular risk factors including obesity, hypertension, dyslipidaemia and physical inactivity [7]. Nevertheless, observational studies comparing T1DM and T2DM suggest that T2DM, when similar to T1DM in terms of control and age of onset, is associated with an overall higher number of complications, especially microvascular and cardiovascular [8,9].

T1DM patients have been found to have a life expectancy at birth of 68.6 years, which was 12.2 years less than in the general population in an Australian cohort [10]. Insulin replacement therapy is the cornerstone treatment for T1DM, but it requires multiple daily injections and glycaemia measurements, with careful planning of time and composition of meals, and a significant detrimental effect on the quality of life [11]. Once β-cells are lost, no oral drug can compensate for insulin production deficiency. Therefore, many efforts have been made to find a curative option: several trials involving the administration of anti-inflammatory drugs have been designed, with the aim of stopping and hopefully reverting the β-cell destruction process, but without substantial success [12]. Pancreatic islet transplantation has been tested, but it requires a surgical procedure and lifelong immunosuppression, and usually T1DM relapses soon due to the destruction of the transplanted islets [13]. The last, intriguing alternative was cell therapy; indeed, in this manuscript, we will review the attempts made so far in treating T1DM with hematopoietic stem/progenitor cell (HSPC) therapy. Based on the experience gathered by the trials designed in the last two decades, we will also discuss the best patient candidate for tailored treatment.

## 2. Hematopoietic Stem/Progenitor Cells for Cell Therapy

HSPCs are self-renewing and multipotent cells that reside in specialized niches within the bone marrow. Identified by the CD34 surface antigen, whose function is still unknown, they are capable of differentiating in all types of blood cells, both of myeloid and lymphoid lineage, and to reconstitute the whole hematopoietic system after bone marrow ablation [14,15]. A small number of CD34^+^ cells can also be found in the peripheral blood (~3 cells/µL), from which they move throughout the body [16]. The physiological significance of their migration seems to correlate with the patrolling of peripheral organs, in which they maintain tissue homeostasis and regeneration, and immune responsiveness [17]. The CD34 marker, although routinely used to identify and isolate human HSPCs, is also expressed by a broader group of cell populations, including multipotent mesenchymal stem/stromal cells (MSCs), vascular endothelial progenitor cells and epithelial progenitor cells [18,19,20,21]. The isolation of CD34^+^ cells from peripheral or bone marrow blood yields a mixture of cells characterized by different states of differentiation, including cells with vasculotrophic function, namely endothelial progenitor cells (EPCs). The discovery of this cell population, initially identified by double positivity for CD34 and KDR markers, by Asahara at the end of the 1990s pioneered over 20 years of research in stem cell biology and regenerative medicine [22]. Since then, numerous studies have attempted to characterize EPC origin and phenotype. As reviewed in more detail elsewhere [17,23,24], two different approaches have been used so far: one aiming at the identification of circulating EPCs by flow cytometric assay of peripheral blood samples, and the other based on cell culture methods. This latter led to the identification of early EPCs (CD45^+^, CD14^+^ CD31^+^ CD34^−^ CD146^−^) and late EPCs (CD31^+^, CD146^+^, CD105^+^, CD45^−^, CD14^−^), whose effective existence and function in vivo are elusive. Overall, both approaches led to controversial results and a general lack of consensus. Today, due to the impossibility of physically separating EPCs from HSPCs for their overlapping phenotype, scientists are inclined to identify circulating EPCs with the more generic CD34^+^/CD133^+^ HSPC population because they are ancestors of EPC [23,25]. In this regard, CD34^+^ cells as a whole are known to possess vascular regeneration capacity and proangiogenic potential, and their circulating level reduction is linked to poorer outcomes in cardiovascular diseases, in chronic haemodialysis patients and after cerebral infarction [16,26,27,28]. Nevertheless, most of the preclinical and clinical studies so far reported were performed in view of the putative cardiovascular protective and pro-angiogenic role of HSPCs, overlooking that these cells are precursors of immune system cells and that possess an immunoregulatory function. Consistently, allogeneic bone marrow HSPCs transplantation in non-obese diabetic (NOD) mice has been shown to prevent diabetes onset and to restore insulin independence (IID) in type 1 diabetic mice, despite not being able to regenerate lost pancreatic islets. Such evidence, together with the fact that transplanted islets from the same allogeneic donor could be accepted by diabetic recipients previously transplanted with bone marrow and transiently restore IID, suggested that the main mechanism of action of HSPCs lies in their immunomodulatory properties rather than a mere regenerative effect. This hypothesis was further supported by clinical evidence that CD34^+^ cells exhibited immune system resetting and reconstitution properties after autologous nonmyeloablative (as opposed to standard peripheral blood stem cell transplantation, which generally implies bone marrow ablation of the host [29]) hematopoietic stem cell transplantation (AHST): in multiple sclerosis studies, AHST determined an increase in thymus-derived naive T cells, a decrease in central-memory T cells, the recovery of a different T-cell receptor repertoire and immune system drifting towards a more tolerant phenotype [30,31]. Similar evidence of the immunotolerant properties of HSPCs was found when assessed for the treatment of systemic lupus erythematosus [32].

Besides CD34^+^ HSPCs, other cell types have been tested in trials for the treatment of T1DM. These include the unselected bone marrow-derived mononuclear cell (BM-MNC) fraction and mesenchymal stem cells (MSCs). The former consists of a pool of different kinds of cells, typically obtained by bone marrow aspiration from the iliac crest, in which CD34^+^ cells represent a fraction of about 0.5–6% and are most likely responsible for the therapeutic effect [33,34]. On the other hand, MSCs are multipotent cells that can differentiate in several cell types, such as osteoblasts, chondroblasts, myocytes, adipocytes and β-cell-like cells [17,35,36,37]; can be collected from various tissues, including bone marrow, fat and umbilical cord blood; and possess immunomodulatory properties and the potential to protect pancreatic islets and promote their regeneration [38,39,40].

One last cell type, i.e., embryonic stem cells (ESCs), is under evaluation in some clinical trials (NCT02239354, NCT04678557, NCT03163511, NCT05210530, NCT04786262). Unlike other cell types, these are manipulated to obtain pancreatic endodermal cells that can be administered to patients to restore insulin production directly [41]. Furthermore, studies with immunodeficient mice have shown their capability to correctly differentiate to stem cell-derived β-like cells and become insulin-sensitive when transplanted [42]. Being allogeneic by definition, they require host immunosuppression, to be engineered to evade immune response, or to be encapsulated in subcutaneous immunoisolation devices [43]. Another downside of these cells is that they are not available worldwide due to ethical concerns. Attempts have been made to reproduce them with patient-derived induced pluripotent stem cells (IPSCs), which have proved a useful model for research but are still unsuitable for therapeutic use due to potential instability [44].

Nevertheless, HSPCs among all cell types have given the largest body of promising results in the treatment of T1DM so far [45,46,47]. Therefore, trials involving these cells (summarised in Table 1) will be our focus.

## 3. Hematopoietic Stem/Progenitor Cells in Clinical Trials

### 3.1. Autologous Nonmyeloablative Hematopoietic Stem Cell Transplantation

The usefulness of HSPCs for the treatment of T1DM has been evaluated in several clinical trials employing the AHST approach. Since T1DM is an autoimmune disease characterized by autoreactivity against pancreatic islet β-cells, with consequent impaired insulin production [2], the researchers aimed to verify if AHST could interrupt or at least decelerate the β-cell destruction by the immune system in a setting of new-onset/early T1DM, with benefits in terms of independence from insulin administration or a reduction in dose [31]. This approach had the potential to prove a more effective, less risky and expensive, and lifelong immunosuppression-free curative alternative to pancreatic islet transplantation [13].

The typical protocol design was rather consistent among the different clinical trials. Generally, it included a stem cell mobilization phase with cyclophosphamide and granulocyte colony-stimulating factor (GCSF), and consequent leukapheresis with a continuous-flow blood cell separator for the isolation of CD34^+^ cells, followed by a conditioning (immune ablative) phase with cyclophosphamide and antithymocyte globulin. At the end of the conditioning treatment, the patients underwent stem cell infusion and antimicrobial prophylaxis in an isolated environment. All protocols were approved by local regulatory authorities/ethical committees.

### 3.2. The Brazilian Study

The first study was conducted by Voltarelli et al. in Brazil (NCT00315133). They enrolled 15 patients, including Black, White, and mixed races with T1DM diagnosed within the previous 6 weeks between November 2003 and July 2006, with a follow-up of 7 to 36 months. Stem cells were mobilized with cyclophosphamide and GCSF; afterwards, the patients underwent leukapheresis, and the cells were frozen in 10% dimethyl sulfoxide in a rate-controlled freezer and stored in the vapor phase of liquid nitrogen. The conditioning phase consisted of a 5-day treatment with cyclophosphamide and rabbit antithymocyte globulin; prophylaxis of the antithymocyte globulin reactions was performed with dexchlorpheniramine, except for patient n°1 (treated with corticosteroids due to diabetic ketoacidosis = DKA). After conditioning, stem cell infusion (at least 3 × 10^6^/kg) was delivered, followed by GCSF administration after 5 days. The patients were isolated in rooms equipped with high-efficiency particulate air filters and received antimicrobial prophylaxis. Exclusion criteria were positive serology for human immunodeficiency virus, hepatitis B or C, pregnancy and underlying hematologic, nephrological, cardiac, psychiatric, or hepatic disease and DKA (see below).

The study gave very promising results: all patients except for n°1 (diagnosed with DKA) achieved IID at a certain point of the follow-up (which lasted for a median of 14.8 months and a maximum of 35 months); thus, thereafter, DKA was listed among the exclusion criteria. Thirteen patients became continuously IID and one resumed insulin treatment 1 year after ASHT. Moreover, the mean area under the curve (AUC) of C-peptide levels before transplantation (92.0 ng/mL per 2 h) showed a statistically significant increase at 6 months, 12 and 24 months; anti-glutamic acid decarboxylase antibody (GADA) levels were significantly lower after 6 months; at the beginning of the study, 11 of 14 patients presented glycated haemoglobin (HbA1c) values above 7%, but in 3 months a persistent drop was observed except for one patient, who eventually relapsed [31].

After ASHT, mild side effects were observed in most patients (such as febrile neutropenia, nausea, vomiting, alopecia), plus a case of bilateral pneumonia that required supplementary oxygen therapy and responded completely to broad-spectrum antibiotics. Further, during follow-up, there was a case of autoimmune hypothyroidism and transient renal dysfunction associated with rhabdomyolysis and a case of mild hypergonadotropic hypogonadism. No mortality was associated with the treatment.

This study provided the first clinical evidence of sustained recovery of IID in T1DM patients after treatment with AHST. Two years later, the same group published the results of the extended follow-up with the addition of eight new patients, for a total of 23 [48]. After a follow-up of 7–58 months, 12 had become continuously IID and 8 only transiently, among which 4 resumed insulin after an upper respiratory tract infection; interestingly, two patients recovered IID after the addition of the dipeptidyl peptidase 4 inhibitor sitagliptin, with the restoration of β-cell function witnessed by the upturn of C-peptide levels. The authors suggested that the beneficial effects of sitagliptin could be due to the rapid suppression of glucagon levels in parallel with further increase in insulin production, together with a potential immunoregulatory function in autoimmune insulitis [48,56]. Unfortunately, the combination of AHST and sitagliptin has not been further tested in other studies. Only three patients never became IID, two of which developed DKA and two underwent corticosteroid treatment. HbA1c levels in continuously IID patients constantly remained under 7% and AUC of C-peptide levels increased significantly in all patients with at least transient IID. Concerning adverse reactions, an additional patient developed bilateral nosocomial pneumonia that effectively responded to intravenous broad-spectrum antibiotics, three patients developed late endocrine dysfunction (autoimmune hypothyroidism, Graves’ disease and transient hypergonadotropic hypogonadism), and nine patients developed oligospermia. No mortality was documented [48].

In summary, Voltarelli et al. confirmed that AHST was capable of reversing T1DM in humans, at least for up 4 years and with an acceptable burden of adverse effects.

### 3.3. The Polish Study

A subsequent study was performed by Snarsky et al. in Poland, initially on a small cohort of eight patients [49]. The Brazilian study protocol was modified by implementing 2–3 preliminary plasmapheresis sessions to remove circulating antibodies and immunological complexes, based on the observation that plasmapheresis can change the clinical course of diabetes [57]. All patients became IID after AHST, and only one resumed insulin treatment later; six patients were given acarbose additionally to improve glycaemic control. After AHST, HbA1c mean levels significantly dropped, and C-peptide levels rose. There were no major complications and some mild adverse effects such as nausea and fever [49].

The cohort of patients was later expanded to 24 participants: 20 remained insulin-free for at least 9.5 months, and 4 of them were still IID at the end of follow-up (up to 80 months). Unfortunately, four patients developed important antithymocyte globulin-related skin reaction/vasculitis, one patient developed pulmonary emphysema after the insertion of a central venous catheter and one patient died from *Pseudomonas Aeruginosa* sepsis [50].

### 3.4. The Chinese Studies

Two different Chinese clinical trials have been officially registered (NCT01341899 and NCT00807651), involving patients recruited in Shanghai and Nanjing; however, multiple papers have been published by the authors, presenting the outcomes of different pools of patients with sometimes very similar baseline characteristics. For this reason, a clear assessment of their overall findings is difficult to perform.

Li et al. recruited 13 Chinese patients and designed a protocol similar to the Brazilian one [51]. Only three patients developed IID, one of which relapsed after 7 months; eight patients simply required reduced insulin doses for adequate glycaemic control and two patients were non-responders. The reason for a lower IID rate compared to the previous studies was ascribed to an average longer time from T1DM onset (up to 12 months), a more aggressive disease (10 patients experienced DKA, although among them were two of the three patients who achieved IID) and stronger immunity (more than 50% of patients developed autoantibodies against two β-cell antigens before AHST). Nevertheless, the levels of serum fasting and post-prandial C-peptide in responding patients for at least 6 months after AHST were significantly higher than before treatment, while plasma HbA1c levels were significantly lower. Only mild side effects were documented, plus a case of autoimmune thyroiditis 6 months after AHST. The researchers also characterized the immunological state of the patients after AHST and found out that only CD4^+^ T lymphocytes, unlike other cells, remained persistently lower after immune reconstitution; the concentrations of serum IL-1, IL-17 and TNF-α at 3 months and TNF-α and TGF-β at 6 months after AHST were significantly lower than before treatment, while TGF-β levels raised at 36 months. Moreover, the number of infused CD34^+^ cells was positively correlated with the concentrations of serum IL-10, IL-4 and TGF-β but negatively with TNF-α (despite IL-10 and IL-4 levels remaining globally unchanged over time). Four out of seven GADA^+^ patients became persistently negative, and another three cases became only transiently negative after AHST; six islet cell antibodies (ICA)^+^ patients became either transiently or continuously ICA-. Only two cases developed insulin autoantibodies (IAA) during follow-up. These findings were consistent with the reconstitution of a tendentially anti-inflammatory environment after AHST [51].

Another Chinese study aimed to clarify the impact of a history of DKA on the outcome of AHST: Gu et al. enrolled 28 patients with a recent T1DM diagnosis (up to 26 weeks before), 11 of which presented with DKA at diagnosis. After AHST, only three patients with previous DKA achieved at least transient IID, versus 12 patients without previous DKA (*p* = 0.051). Moreover, the DKA patients showed a delayed response to AHST and trended towards a higher insulin dose requirement after 1 year, while in the other patients, the ongoing destruction of the remaining β-cells slowed or stopped, and the fasting C-peptide concentration and AUC increased significantly [52].

Zhang et al. found no significant difference in immune cell populations between six patients who became insulin-free after AHST and three patients who remained dependent, either pre-AHST or after 6 months; they also performed an array-based genomic study and analysed the transcriptome in the peripheral blood mononuclear cells of these patients (PBMC) [53]. They discovered that most of the immune-related genes were upregulated in both groups, except for regulatory genes such as FoxP3 and IL-10, leading to the speculation that the beneficial effects of AHST could be due to the deletion of autoreactive clones rather than the activation of immunoregulatory response. Furthermore, they identified two major gene expression pathways: the selective expression of chemokine receptors during T-cell polarization, and IL-12 and Stat4-dependent signalling pathways in Th1 development. This finding suggests a predominant differentiation of T lymphocytes in Th1 type after AHST and possibly with a new T-cell receptor (TCR) repertoire, as is consistent with observations in former systemic sclerosis studies [31,53,58].

In 2014, D’Addio et al. performed a pooled analysis of the results of the trials conducted by Snarsky et al., Li et al. and Gu et al. (65 patients in total) [50,51,52,59]. Overall, 59% of patients accomplished IID within the first 6 months after treatment, while only 32% were still IID at the end of their follow-up. There was no significant correlation between HbA1c and C-peptide levels and the number of CD34^+^ cells; a multivariate analysis was negative for sex, age and baseline HbA1c and C-peptide levels. However, the authors found out that patients treated earlier (within 6 weeks of the onset of T1DM) were twice as likely to achieve IID (82% versus 40%) [59].

In 2017, Gu et al. published the results of a phase II non-randomized trial involving 20 patients treated with AHST (four patients withdrew their consent, and one was lost to follow-up) and 20 patients regularly treated with insulin injections (four were lost to follow-up), with a disease duration of fewer than 6 months. Fourteen patients in the AHST branch became IID, three of which were up to the end of follow-up (48 months), while only one patient in the insulin treatment branch became transiently IID and then relapsed. The adverse event rate was in line with the other studies and, interestingly, there was no substantial difference between the two branches in terms of the development of endocrinopathies: two patients were diagnosed with Graves’ disease after AHST, while among insulin-treated patients, two developed Graves’ disease and one developed autoimmune hypothyroidism [55].

Finally, Ye et al. compared immune responses after AHST versus insulin-only therapy, reporting reduced proportions of Th1 and Th17 cells, and IFN-γ, IL-2, IL-12p40 and IL-17A levels in the PBMC culture supernatants from these patients; they also found increased IL-10, TGF-β and Foxp3 mRNA expression but no significant Treg cell expansion [60].

### 3.5. The Mexican Study

Due to the well-known potential adverse reactions related to immune ablation, Cantú-Rodríguez et al. designed a simplified AHST protocol contemplating the use of fludarabine instead of antithymocyte globulin and a lower dose of cyclophosphamide for conditioning. Of note, the subjects enrolled (16 patients less than 3 months from T1DM diagnosis) were managed in an outpatient setting: the aim was to reduce toxicity, limit the cost of the procedure and lower the risk of nosocomial infections. No serious adverse event was documented, but 52% of patients experienced side effects: the most common included nausea, vomiting, fever and alopecia; four patients developed neutropenic fever, but none required hospitalization, and they were all managed with an oral amoxicillin regimen; one patient experienced haemorrhagic cystitis that was resolved in less than 36 h with intense hydration. Outcomes were comparable to other studies, with seven patients (44%) achieving and maintaining complete IID and six (37%) achieving only partial IID [54].

## 4. Other Cell Therapy Approaches in Clinical Trials

The infusion of different types of stem cells/stem cell-enriched blood for the treatment of T1DM has been attempted, with heterogeneous results and an overall lower efficacy profile than HPSCs [45,46,47]. Nevertheless, the general mechanism of action appears to be similar, i.e., immunomodulation rather than β-cell regeneration. A brief summary of the most relevant examples is reported here for completeness.

Autologous BM-MNCs were tested in a very small trial, with two patients in the treatment arm and one control with standard therapy, with a diagnosis of diabetes not older than 60 days. Bone marrow was stimulated with GCSF for 4 days and then collected on the fifth. The authors documented negativization of antibody levels, increased levels of C-peptide and decreased blood glucose and HbA1c, with a lower daily insulin dose required compared to the standard therapy patient [61].

The transfusion of autologous umbilical cord blood has been tested in children without substantial success [62,63,64,65]. On the other hand, allogeneic umbilical cord MSCs were tested against standard therapy in two different trials, alone with a second infusion after 3 months [66] or combined with autologous BM-MNCs [67]. Both showed a good safety profile and improvement in metabolic parameters, the former allowing 3/27 patients to achieve IID lasting up to 12 months.

In addition, the infusion of allogeneic Wharton’s jelly-derived MSCs from the umbilical cord in a small double-blind study gave encouraging results, with 3/15 patients in the treatment group discontinuing insulin after 21 months, an overall improvement of the laboratory parameters and lowering of the required insulin dosage [68]. Other clinical trials have been designed with Warton’s jelly-derived MSCs [69], but there are no published results to our knowledge to date.

An Iranian group has also attempted allogeneic transplantation of foetal liver-derived stem cells in three different trials, with an overall improvement of metabolic parameters and the achievement of IID in three patients for up to 3 months [70,71,72].

Autologous bone marrow-derived MSCs infusion was tested against insulin therapy in a 20-patient study, showing preserved or improved C-peptide peak values and C-peptide AUC in the first year in the treatment arm [73].

Another group tested, in a non-controlled study, the intraportal infusion of a combination of allogeneic adipose tissue-derived insulin-secreting MSCs and BM-MNCs in 11 patients, after a nonmyeloablative low-intensity conditioning phase involving the irradiation of lymph nodes, spleen, part of the pelvic bones, and lumbar vertebrae, as well as the administration of Anti-T cell antibody and Anti-B cell antibody. They documented a decreased exogenous insulin requirement and HbA1c values, increased serum C-peptide levels and freedom from DKA events [74]. They subsequently designed a second study to compare allogeneic vs autologous cell infusion and observed better metabolic parameters in the autologous group [75]. Unfortunately, this trial lacked a standard therapy control.

## 5. The Profile of the Responder Patients

Although the clinical studies presented above were rather homogeneous in terms of AHST protocol, sometimes with just small variations, there was a significant difference in outcomes, especially between the Brazilian and Polish studies and all the others. Many factors could contribute to explaining this, including the pre-treatment duration of the disease, the history of DKA, the HLA haplotype, the insulin dose before treatment and the ethnicity of the patients. In the attempt to identify the characteristics associated with a better outcome after AHST, we pooled the patients from the different trials and performed a multiple logistic regression analysis (RStudio version 2022.12.0+353) to determine the most likely contributors in the achievement of at least transient IID after AHST, and in maintaining IID for at least 12 months. Due to significantly similar baseline characteristics shared by some of the patients enrolled in the different Chinese studies, in order to avoid potential duplication, only the first study published by Gu et al. [52], which included the highest number of AHST-treated patients, was considered.

Our final population accounted for 90 patients; due to the low sample number, we only selected the variables available for all the population (sex, age, body mass index, history of DKA and time from diagnosis to treatment). The time from diagnosis to treatment was transformed in a binomial variable (≤/> 12 weeks) due to the lack of accurate information in some of the studies. Characteristics of the patients can be found in Table 2.

**Figure 1:**
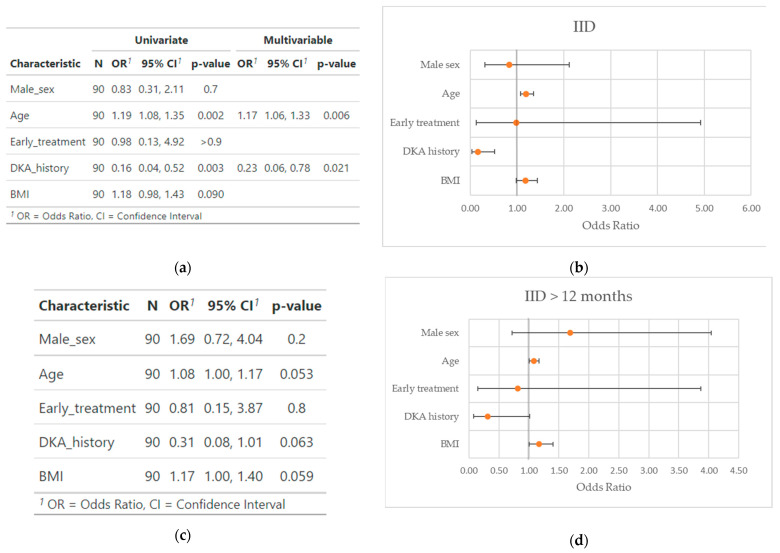
Odds ratio, 95% confidence interval and statistical significance of the patient characteristics for the achievement of insulin independence (**a**) and maintaining it for >12 months (**c**); respective forest plots (**b**,**d**). Forest plots represent the results of the univariate analysis.

We performed a univariable analysis and then built a multivariable model with the characteristics we found to be significant during the analysis (Figure 1).

As a result, we found a significantly higher probability of achieving IID in older patients (OR 1.17, 95%CI 1.06–1.33, *p* = 0.006) and a significantly lower probability in patients with a history of DKA (OR 0.23, 95%CI 0.06–0.78, *p* = 0.021). We did not find a significant interaction between the covariates (*p* for interaction = 0.18). We then verified the model fit with the Hosmer and Lemeshow goodness of fit test (*p* = 0.73).

Conversely, we did not find any significant statistical association between the probability of maintaining IID for at least 12 months and any of the variables tested, with just a non-significant trend towards a higher probability for older age (OR 1.08, 95%CI 1.00–1.17, *p* = 0.053) and higher BMI (OR 1.17, 95%CI 0.08–1.01, *p* = 0.059), and a lower probability in DKA history patients (OR 0.37, 95%CI 0.09–1.32, *p* = 0.14); therefore, in this case we did not build a multivariable model.

## 6. Discussion

What have we learnt from these trials? Is the recovery of IID with AHST a realistic objective to date? Although the current evidence propends for an overall efficacy of AHST in terms of insulin requirement, C-peptide concentration and HbA1c levels, as already assessed in multiple metanalyses [45,46,47], several limitations make it challenging to implement in clinical practice. First, the scientific evidence behind the trials is low quality: all trials were open-label, and only one trial included a control group [55], but it was non-randomized. Although this may have had a lesser impact on objective parameters such as C-peptide concentration, it could have influenced the choice to declare a patient insulin-independent or the insulin dose to administer. Second, it is hard to draw overall conclusions from the Chinese studies due to the similarity of the baseline characteristics in some patients. Third, AHST is a demanding treatment for the patient, requires transient immunosuppression and could potentially lead to serious adverse events (there was a death during the Polish study due to P. Aeruginosa sepsis). Fourth, improving the patient quality of life, with the long-term restoration of IID, would probably be the only achievement that could make it worth the effort, unless future data prove a prognostic benefit of AHST. Although many patients have been observed to achieve IID after the treatment, far fewer have been able to continue this until the end of follow-up.

What should be done to increase the feasibility and appeal of AHST? First, the selection of the ideal candidates for this treatment would improve its overall efficacy. Our findings support an important role of a previous episode of DKA in determining a lower probability of restoring IID after treatment; nevertheless, this was already intuited by Voltarelli et al. More interesting is the significantly higher number of patients who achieved IID we found associated with older age: this could be explained by a higher severity of the disease when it manifests in the younger population. In fact, younger children progress more rapidly than older children and adults from antibody positivity to T1DM; moreover, in children diagnosed before 7 years, the percentage of insulin-containing islets with evidence of insulitis is much higher than in older teenagers (>75% vs. 25%) [76].

Unfortunately, current data are not exhaustive enough to determine a comprehensive profile of patients that are more likely to respond. The low overall number of patients, the heterogeneity in data available from the different trials and the significantly different outcomes were the major limitations to our analysis. The relationship between the outcome and the immunophenotype would have been very interesting to observe but, unfortunately, we only had partial data for the HLA haplotypes of the patients, and a statistical analysis would have been impossible to perform. Inflammation and infections could have played significant roles as well, but these we were unable to describe accurately: for example, some patients lost IID after upper respiratory tract infections, suggesting that the autoimmune process could be awakened by inflammatory/infectious stimuli. In addition, although we could not find any statistically significant association between the time from diagnosis and the probability of achieving and maintain IID, which the need to transform the variables in binomial has probably contributed to, a shorter time from the insurgence of the disease likely remains one of the most determinant factors, for physio-pathological reasons: the sooner we stop the autoimmune process of β-cell destruction, the more will be left to produce endogenous insulin. AHST does not induce pancreatic islet regeneration [77], and its potential to restore IID supports a significant role of inflammation in β-cell dysfunction [76], which is capable of reducing the production of insulin beyond the mere effect of β-cell destruction, therefore generating a quota of insulin production impairment that is indeed reversible. Ideally, even better would be intercepting T1DM at its earlier stages, before its clinical manifestation. Screening tools are being developed [78], and this could prove feasible in the future. The potential role of the combination with drug therapy deserves to be further assessed as well: Couri et al. documented a second restoration of IID after relapse in two patients following the administration of sitagliptin, but this has not been explored further [48]. Moreover, stronger scientific evidence is required, with the design of a solid phase II randomized controlled trial, although blinding could be unfeasible with the immune ablation step. Finally, avoiding immune ablation could reduce the risks and costs of this treatment. The necessity for immune ablation itself needs to be verified, since we have learnt from trials involving other kinds of stem cells that it might not be required to restore IID [66,68,71].

## 7. Conclusions

In conclusion, although the efficacy of AHST in the treatment of T1DM has been assessed in multiple clinical trials, several limitations hamper the implementation of this treatment in routine clinical practice. Selecting the ideal candidates for AHST, with the highest chances of obtaining long-lasting IID, could prove crucial, but very little evidence exists on the profile of these patients and further studies are required to address this question.

## Figures and Tables

**Table 1:** Clinical trials testing AHST in the treatment of T1DM.

Study	Population	Study Design	Outcomes	Adverse Events
[31,48]	Twenty-three pts (12–35 y.o.), diagnosis of T1DM within the previous 6 weeks. Only 1 DKA patient.	Phase I/II open-label clinical trial. Immune ablation with cyclophosphamide and ATG, followed by i.v. infusion of autologous CD34^+^ cells (10.52 × 10^6^ cells/kg) and GCSF.	Most pts showed a reduction in HbA1c levels and an increase in C-peptide levels after treatment. Twenty pts experienced IID (12 until the end of follow-up, up to 4 yrs).	Bilateral nosocomial pneumonia (2 pts), posttransplant oligospermia (9 pts), Graves’ disease (1 pt), transient hypergonadotropic hypogonadism (1 pt), autoimmune hypothyroidism (1 pt).
[49,50]	Twenty-four pts (12–35 y.o.), diagnosis of T1DM within the previous 6 weeks, sustained endogenous secretion of insulin and WHO performance status ≤ 2. No history of DKA.	Phase II open-label clinical trial. Preliminary plasmapheresis, then immune ablation with cyclophosphamide and ATG, followed by i.v. infusion of autologous CD34^+^ cells (4.19 × 10^6^ cells/kg) and GCSF.	General reduction in HbA1c levels and increase in C-peptide levels after treatment. Twenty pts achieved IID insulin (4 until the end of follow-up, up to 80 mo).	ATG-related skin reaction/vasculitis (4 pts), neutropenic fever (12 pts), sepsis (4 pts, out of which 1 was fatal).
[51]	Thirteen pts (<25 y.o.) symptom insurgence within 12 months and positive for at least 1 between GADA, IA-2A, ICA, IAA.	Open-label study. Immune ablation with cyclophosphamide and ATG, followed by i.v. infusion of autologous CD34^+^ cells (2.05–9.60 × 10^6^/kg) and GCSF.	Eleven pts exhibited increased levels of C-peptide and required a significantly reduced dose of insulin after AHST, 3 of which achieved and maintained IID for 7 months, more than 3, or 4 y, respectively. HbA1c levels normalized in 7/8 pts.	Mild side effects (cytotoxic drug-related nausea, vomiting, fever, alopecia), 1 case of sub-clinical hypothyroidism.
[52]	Twenty-eight pts (14–27 y.o.), recent diagnosis of T1DM with time from symptom onset to AHST 4–26 weeks.	Phase II open-label clinical trial. Immune ablation with cyclophosphamide and ATG, followed by i.v. infusion of autologous CD34^+^ cells.	Fifteen pts achieved IID (7 relapsed). General decrease in HbA1c and GADA and increase in C-peptide levels.	Most patients experienced febrile neutropenia, nausea, vomiting, alopecia, bone marrow suppression, Graves’ disease (1 pt), hypothyroidism (1 pt).
[53]	Nine pts (15–25 y.o.) diagnosed with T1DM within 6 months and GADA positivity. No DKA.	Open-label study. Immune ablation with cyclophosphamide and ATG, followed by infusion via peripheral vein of autologous CD34^+^ cells.	Six pts achieved IID with increase in C-peptide levels. HbA1c and GADA levels dropped in 8 pts.	Staphylococcus and streptococcus infection (4 pts), vulvovaginal candidiasis (1 pt).
[54]	Sixteen pts (8–25 y.o.) diagnosed with T1DM within 3 months and GADA positivity.	Open-label study. Immune ablation with cyclophosphamide and ATG, followed by infusion via peripheral vein of autologous CD34^+^ cells (mean 11.5 × 10^6^/kg).	Reduction in HbA1c levels and insulin dose in 13 patients, 7 of which achieved IID. General reduction in GADA titres.	Mild side effects (nausea, vomiting, fe-ver, alopecia), neutropenic fever (4 pts), haemorrhagic cystitis (1 pt).
[55]	Forty pts (14–27 y.o.), recent diagnosis of T1DM with time from symptom onset to AHST 4–26 weeks.	Phase II, parallel-assignment, non-randomized clinical trial. Treatment group pts underwent immune ablation with cyclophosphamide and ATG, followed by i.v. infusion of autologous CD34^+^ cells. Control group pts received regular insulin therapy.	Increase in C-peptide levels in treatment group and decline in control group at 48 mo. Comparable reduction in HbA1c levels in both groups. Fourteen pts in treatment group experienced IID (3 until the end of follow-up, up to 48 mo). One pt in control group experienced transient insulin independence for 7 mo.	Graves’ disease (2 pts on treatment, 1 pt in control group), autoimmune thyroid disease (2 pts in control group).

Abbreviations: ASHT = autologous hematopoietic stem cell transplantation; ATG = anti-thymocyte globulin; DKA = diabetic ketoacidosis, HbA1c = glycated haemoglobin; GADA = glutamic acid decarboxylase antibody; GCSF = granulocyte colony stimulating factor; IAA = insulin autoantibody; IA-2A = protein tyrosine phosphatase antibody; ICA = islet cell antibody (ICA); IID = insulin independence; pt = patient; T1DM = type 1 diabetes mellitus; y.o. = years old; WHO = World Health Organization.

**Table 2:** Characteristics of patients enrolled in the clinical trials selected for statistical analysis.

Study	Population (n)	Male Sex (n)	Age (y)	Early Treatment * (n)	DKA History (n)	BMI (kg/m^2^)	IID (n)	IID > 12 Months (n)
[48]	23	17	18.4 (4.6)	23	1	19.7 (2.2)	22	15
[50]	23 †	16	24.8 (4.6)	24	0	20.8 (1.6)	22	17
[52]	28	14	17.6 (3.8)	21	11	18.7 (1.9)	15	8
[54]	16	9	12.0 (2.6)	16	3	19.6 (2.7)	7	7

* Within 12 months after diagnosis. † One patient died shortly after AHST due to treatment complication; therefore, they were not included in the statistical analysis. Age and BMI are presented as mean (standard deviation). Abbreviations: BMI = body mass index; DKA = diabetic ketoacidosis; IID = insulin independence.
